# Public perspectives on the use of different data types for prediction in healthcare

**DOI:** 10.1093/jamia/ocae009

**Published:** 2024-02-01

**Authors:** Paige Nong, Julia Adler-Milstein, Sharon Kardia, Jodyn Platt

**Affiliations:** Division of Health Policy and Management, University of Minnesota School of Public Health, Minneapolis, MN 55455, United States; Division of Clinical Informatics and Digital Transformation, University of California San Francisco Department of Medicine, San Francisco, CA 94143, United States; Department of Epidemiology, University of Michigan School of Public Health, Ann Arbor, MI 48109, United States; Department of Learning Health Sciences, Michigan Medicine, Ann Arbor, MI 48109, United States

**Keywords:** predictive models, trust, public opinion, data collection

## Abstract

**Objective:**

Understand public comfort with the use of different data types for predictive models

**Materials and Methods:**

We analyzed data from a national survey of US adults (*n* = 1436) fielded from November to December 2021. For three categories of data (identified using factor analysis), we use descriptive statistics to capture comfort level.

**Results:**

Public comfort with data use for prediction is low. For 13 of 15 data types, most respondents were uncomfortable with that data being used for prediction. In factor analysis, 15 types of data grouped into three categories based on public comfort: (1) personal characteristic data, (2) health-related data, and (3) sensitive data. Mean comfort was highest for health-related data (2.45, SD 0.84, range 1-4), followed by personal characteristic data (2.36, SD 0.94), and sensitive data (1.88, SD 0.77). Across these categories, we observe a statistically significant positive relationship between trust in health systems’ use of patient information and comfort with data use for prediction.

**Discussion:**

Although public trust is recognized as important for the sustainable expansion of predictive tools, current policy does not reflect public concerns. Low comfort with data use for prediction should be addressed in order to prevent potential negative impacts on trust in healthcare.

**Conclusion:**

Our results provide empirical evidence on public perspectives, which are important for shaping the use of predictive models. Findings demonstrate a need for realignment of policy around the sensitivity of non-clinical data categories.

## Background and significance

Predictive models are used to inform management, administration, and clinical care across the US healthcare system.^1–4^ In 2021, a large majority of acute care organizations were using predictive analytics and risk-based stratification to manage their patient populations.^5^^,^^6^ Predictive models and artificial intelligence (AI)/machine learning (ML) in healthcare rely on increasingly large amounts of diverse data, with the goal of greater precision and more accurate predictive capability to improve patient care. At present, there are few restrictions on the use of de-identified data for these models, often under the presumption that de-identification protects privacy and that patients are comfortable with the risk associated with de-identified data. Data considered sensitive, and therefore subject to higher levels of scrutiny, are limited to a small number of HIPAA-defined identifiers and/or information about disease conditions that could be conceived of as stigmatizing (mental health diagnoses, HIV status) or as inherently identifiable (genomic sequence data). Predictive tools are built using these data on clinical indicators and diagnoses,^7–9^ but they also use data on patient race and ethnicity, employment status, religious affiliation, and history of paying medical bills.^10–12^

Unfortunately, there is not a large body of evidence analyzing how the public understands and responds to data collection for prediction or related informatics tools like AI/ML.^13^ Indeed, patient perspectives are typically analyzed in relation to patient-facing tools like patient portals or apps.^14^^,^^15^ While recent empirical analyses of survey data have examined how people feel about the use of AI, ^16^^,^^17^ this work does not specifically address how they view the use of data that underlie these tools. It is particularly important to assess whether the public is comfortable with the use of specific types of data in the context of prediction because violation of public comfort may threaten trust in the healthcare system and undermine the sustainability of predictive systems. Patients are reporting and managing their data,^18^ and should be engaged as central stakeholders whose perspectives are valued according to federal guidelines and policy guidelines.^19^^,^^20^ These guidelines and some early analysis^13^ center the significance of trust and the ethical obligation to include public perspectives in the design of AI-driven predictive systems.

It is also important to understand the predictors of public comfort to build a high-quality system of prediction in healthcare. Trust in health systems or organizations is an important factor related to patient behavior, with impacts on a variety of indicators of patient engagement and utilization.^21–23^ Certain dimensions of trust play particularly significant roles in relation to information disclosure and attitudes toward information technology.^24^^,^^25^ Prior work has identified that low trust and experiences of discrimination in the healthcare system are related to withholding information from providers, highlighting the stakes of trust in a data-driven healthcare system.^26^^,^^27^ Namely, missing data will lead to poorer quality models. In order to understand the ways public perspectives will shape the future and sustainability of predictive systems, it is necessary to understand the factors that shape their perspectives. For example, familiarity with the health system or having health insurance may increase general comfort,^28^ which may extend to data used in predictive models. Similarly, factors that shape access and attitudes such as health status, income, and education are also likely to be associated with comfort.^16^ Identifying these relationships will allow policymakers and health systems to better understand and account for public comfort as they continue to design policy and practice for prediction.

## Objective

This study examines public comfort with the use of different data types for prediction in healthcare. It also analyzes predictors of comfort with data use, with an emphasis on understanding the role of trust in the healthcare system. The research questions are as follows:

1. Does public comfort vary between different types of data used for prediction in healthcare? What are the underlying dimensions (multivariate factors) of public comfort with use of different data types for prediction in healthcare?
2. What predicts comfort with the use of data for predictive modeling in healthcare?

## Methods

This study uses data from an original cross-sectional survey of US adults who can speak English. The survey sample is drawn from the National Opinion Research Center’s (NORC) AmeriSpeak Panel. Adults aged 21 and older from the AmeriSpeak panel were considered eligible for the survey, which was made available online. African American respondents, Hispanic respondents, and respondents earning less than 200% of the federal poverty level were oversampled to ensure adequate representation and statistical power. For details on the AmeriSpeak panel and sampling details, including survey weights, please see the Supplementary material. Poststratification survey weights calculated based on the Census Bureau’s Current Population Survey were applied to produce nationally representative estimates in the Supplementary analysis.

Based on extensive experience with previous national surveys and focus groups,^26^^,^^28^^,^^29^ the research team produced a 90-s explanatory video describing how health information is used in the US healthcare system to ensure that respondents understood certain aspects of health IT. The expert advisory board for this study, which includes national experts on health informatics and data sharing, reviewed the video and provided input on the content, concepts, and wording. The video was also presented in focus groups to clarify the content and assess the valence of the language used prior to fielding with the national sample. All respondents viewed the final version of this video at the beginning of the survey. Definitions of key terms (health system, healthcare provider, etc.) were provided and available to respondents as hover-over text each time the terms were used in the survey. Predictive models were defined and described in a short paragraph (Flesch-Kincaid score 8.7) immediately preceding the survey questions on prediction in healthcare. The paragraph included popular examples of predictive technology applications outside of and within the healthcare system. This description and the key term definitions are available in the Supplementary material.

**Figure 1.**
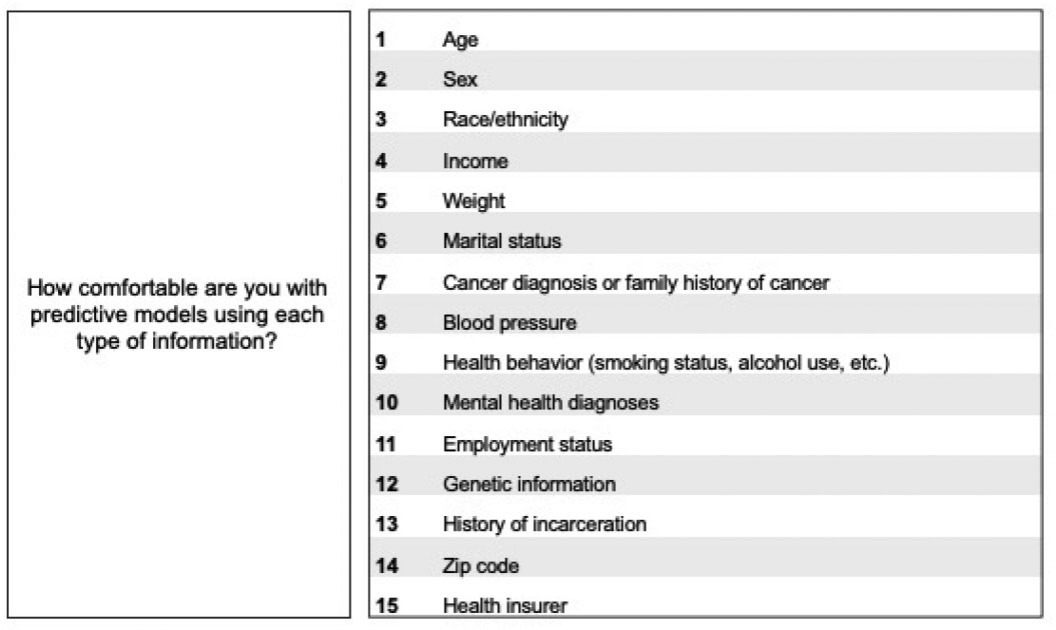
Survey measure: types of data used for prediction.

For each of 15 data types, respondents indicated their comfort with use for predictive models in healthcare. Survey respondents indicated their comfort level on a four-point scale (1 = Not comfortable to 4 = Very comfortable). These data types, ranging from clinical indicators to social determinants of health,^30^ were presented to respondents in randomized order to balance the potential for bias related to item order. All 15 data types presented to survey respondents are listed in Figure 1.

The survey also measured trust in the health system’s competence and integrity with data use. Competence was measured by two survey items using a four-point true scale (1 = Not true, 4 = Very true). Respondents indicated how true it was that their health systems “*have a good track record of using health information responsibly*” and “*can be trusted to keep health information secure”.* These measures reflect trust in system competence with information use. Respondents also reported their perceptions of the health system’s integrity related to information use through multiple survey measures (health systems “*tell me how my health information is used”* and “*would never mislead me about how my health information is used”*).^31^^,^^32^

Lastly, the survey captured demographics including self-reported age in years, sex, and race/ethnicity (Multiracial, Hispanic, non-Hispanic Asian, non-Hispanic Black, non-Hispanic White, and other). Respondents reported their annual household income and education level (no high school diploma, high school or equivalent, some college, BA or more). Health-related questions include health insurance status (insured/uninsured), healthcare utilization in the past 12 months, self-reported health status (poor to very good), and experiences of discrimination in the healthcare system (yes/no).

We tested the survey for comprehension through cognitive interviews (*n* = 17) during which participants “thought aloud” as they took the survey to identify items that were unclear or confusing. The survey was edited based on these interviews, then pre-tested using MTurk (*n* = 550), and pilot tested with a sample of AmeriSpeak panel participants (*n* = 150).

### Analysis

To answer the first research question (identifying multivariate factors of public comfort), distributions of comfort with each of the 15 data types were examined along with their correlations. Exploratory factor analysis was conducted to identify multivariate factors underlying public comfort with the use of all 15 data types. An oblique promax rotation was used. The Kaiser-Meyer-Olkin (KMO) value was 0.95, indicating that sampling adequacy was very strong. Variables were considered to load on a factor when the factor loading was 0.4 or above. Using this criterion, three factors were identified and confirmed with a screen test indicating that three factors should be retained. This produced three factors, which were then confirmed using Cronbach’s alpha (see Supplementary File for complete details).

The three dimensions identified in the factor analysis were named based on the data types that loaded on each factor: personal characteristic data (four types), health-related data (five types), and sensitive data (six types). The three factors were then analyzed as dependent variables in bivariable and multivariable regressions to answer research question two—that is, to identify the predictors of comfort. To do this, composite measures of comfort were calculated for each respondent as the mean comfort score for all data types that loaded on each factor. Bivariable regressions of each data type were conducted on the measures of trust (perceived health system competence and integrity with data use), demographic and health-related covariates (race/ethnicity, income, education, insurance status, self-reported health status, utilization in the last 12 months, experiences of discrimination) listed in the section above. Observations with missing data for demographics, comfort with data use, and the other covariates listed in Table 1 were excluded. This resulted in an analytic sample of 1436 respondents. Multivariable OLS regressions were then conducted for each dependent variable (mean comfort with personal characteristic data, mean comfort with health-related data, and mean comfort with sensitive data types). This study was approved by the institutional review boards of the University of Michigan and NORC. Participants provided written consent and were paid for their time according to NORC’s standards for participant incentives based on the duration of the survey.

**Table 1.** Descriptive statistics (*n* = 1436).

Measure	*n*	%
**Sex**		
Female	718	50
Male	718	50
**Age**		
18-29	87	6.1
30-44	411	28.6
45-59	364	25.4
60+	574	40
**Race/ethnicity**		
White	895	62.3
Hispanic	255	17.8
Black	191	13.3
Multiracial and other	57	4
Asian	38	2.7
**Education**		
Less than high school	37	2.6
High school	242	16.9
Some college	666	46.4
BA or more	491	34.2
**Annual household income**		
<$50 000	727	50.6
At least $50 000	709	49.4
**Health insurance coverage**		
No	94	6.6
Yes	1342	93.5
**Self-reported health**		
Poor to fair	336	23.4
Good	612	42.6
Very good to excellent	488	34
**Last healthcare visit**		
Longer than 1 year	222	15.5
Within past year	1214	84.5
**System competence with data use (mean, SD)**	2.3	0.82
**System integrity with data use (mean, SD)**	2.4	0.9

Observations with missing data were excluded.

## Results

A total of 1541 participants completed the survey. As reported in Table 1, half of the sample self-identified as female. A large majority of respondents had health insurance (93.5%) and had seen a healthcare provider within the previous year (84.5%). The sample was roughly evenly divided between individuals reporting less than $50 000 in annual household income and those earning more than $50 000 per year. We present the unweighted descriptive statistics and multivariable analyses below in order to ensure adequate representation and statistical power for the oversampled populations (African American respondents, Hispanic respondents, and respondents earning less than 200% of the federal poverty level). According to the Census Bureau’s Current Population Survey, our sample was more likely to rent their home than the US adult population. Our sample also had a higher proportion of single adults than the US population. For full details on how this national sample reflects and deviates from the US population according to the Census Bureau’s Current Population Survey, please see the Supplementary material.

**Figure 2.**
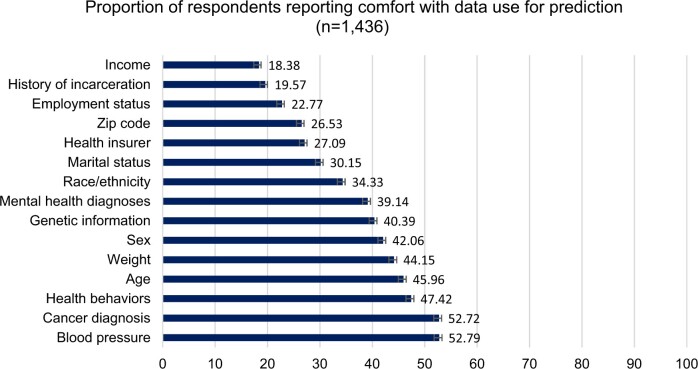
Comfort with 15 data types for prediction in healthcare. In Figure 2, comfort is defined as “very comfortable” or “somewhat comfortable” with the use of each type of data for prediction.

Descriptive statistics indicate that comfort with data use for prediction is low (Figure 2). Only two of the data types analyzed here, blood pressure and cancer diagnosis, were acceptable to a majority of the sample. Comfort with the use of income for prediction in healthcare was lowest (18.38%), followed closely by history of incarceration (19.57%).

*What are the underlying, co-varying dimensions (multivariate factors) of public comfort with use of various data types for prediction in healthcare?*

Exploratory factor analysis revealed the multivariate factors underlying public comfort with the use of all 15 data types. The data types and their factor loadings are listed in Table 2.

**Table 2.** Factor analysis of comfort with the use of all data types for prediction.

Variable	Factor 1	Factor 2	Factor 3
Eigenvalue	8.43	1.34	0.69
Age	**0.79**	0.18	0.005
Sex	**0.80**	0.09	0.01
Race/ethnicity	**0.66**	0.02	0.14
Weight	**0.73**	0.24	0.01
Cancer diagnosis or family history	0.06	**0.89**	0.06
Blood pressure	0.12	**0.88**	0.03
Health behaviors	0.11	**0.67**	0.05
Mental health diagnoses	0.03	**0.69**	0.17
Genetic information	0.17	**0.44**	0.21
Income	0.17	0.06	**0.65**
Marital status	0.38	0.04	**0.49**
Employment status	0.02	0.08	**0.80**
History of incarceration	0.05	0.04	**0.82**
Zip code	0.09	0.02	**0.71**
Health insurer	0.01	0.06	**0.76**

The variable’s factor loading is in bold when it is above the threshold of 0.4.

**Figure 3.**
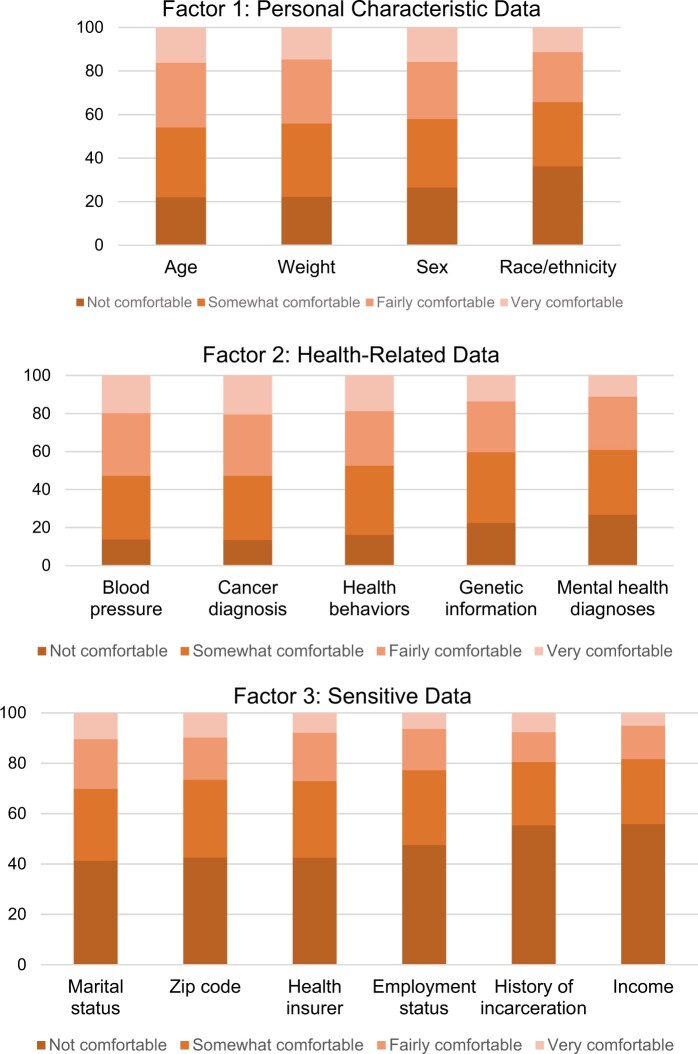
Distributions of comfort with each data type by factor.

The variables that loaded on Factor 1 were largely demographic and personal characteristic data, including age, weight, sex, and race/ethnicity. Variables that met the criteria for loading on Factor 2 included health-related information like health status and diagnoses (eg, blood pressure, cancer diagnosis, health behaviors). Factor 3 included variables describing certain aspects of social experience or position (eg, income, marital status, history of incarceration). Comfort with these data was especially low. For further analysis, these factors are labeled as follows: (1) personal characteristic data, (2) health-related data, and (3) sensitive data. Figure 3 shows the distributions of comfort with each data type according to the factor on which the variables loaded. These figures demonstrate the ways comfort varied according to factor. We observe high proportions of participants reporting low comfort with Factor 3 (sensitive data). We observe a more even distribution of comfort across Factor 1 (personal characteristic data). Discomfort with Factor 2 (health-related data) was generally low, with most participants indicating they were somewhat or fairly comfortable with these data types being used for prediction.

*What individual-level variables predict the multivariate factors of comfort with the use of data for predictive modeling in healthcare?*

Mean comfort with the use of health-related data was highest at 2.45 (SD 0.84, range 1-4). Mean comfort with use of personal characteristic data (2.36, SD 0.94) and sensitive data (1.88, SD 0.77) were lower. Multivariable analysis indicates that trust in system integrity and competence with data use were consistently positively predictive of comfort with use of all three data categories (see Table 3 where coefficients represent fractional differences in average comfort with each factor or data type). Experiences of discrimination in healthcare were statistically significant negative predictors of comfort with the use of personal characteristic data (*b* = −0.13, *P* = .03) and sensitive data (*b* = −0.13, *P* = .008) in the multivariable analysis. This may indicate a concern among people who have experienced discrimination in healthcare that they are vulnerable to digital marginalization or harm based on their social identities.

**Table 3.** Multivariable OLS regression results for each composite measure of comfort.

	Personal characteristic data		Health-related data		Sensitive data	
Variable	*b*	*P*-value	*b*	*P*-value	*b*	*P*-value
Female (ref. male)	−0.21	<.001***	−0.13	.002**	−0.16	<.001***
**Age**						
18-29	Ref		Ref		Ref	
30-44	0.003	.97	−0.16	.07	−0.13	.14
45-59	−0.13	.21	−0.27	.004**	−0.29	<.001***
60+	−0.14	.17	−0.22	.02*	−0.32	<.001***
**Race/ethnicity**						
White	Ref		Ref		Ref	
Black	−0.19	.004**	−0.16	.01*	0.002	.97
Hispanic	−0.02	.71	0.003	.96	0.06	.26
Asian	0.04	.8	0.03	.83	0.15	.2
Other	−0.16	.18	−0.08	.48	0.08	.39
**Education**						
Less than high school	Ref		Ref		Ref	
High school	−0.24	.1	−0.13	.34	−0.03	.8
Some college	−0.18	.2	−0.07	.6	−0.02	.84
BA or more	−0.05	.75	0.04	.73	0.01	.93
**Annual household income**						
<$50 000	Ref		Ref		Ref	
At least $50 000	0.1	.03*	0.1	.02*	−0.03	.45
**Health insurance coverage**						
No	Ref		Ref		Ref	
Yes	0.2	.03*	0.12	.16	0.07	.34
**Experienced discrimination**						
No	Ref		Ref		Ref	
Yes	−0.13	.03*	−0.02	.69	−0.13	.008**
**System integrity with data**	0.08	.01*	0.09	<.001***	0.11	<.001***
**System competence with data**	0.35	<.001***	0.36	<.001***	0.27	<.001***

Statistically insignificant results for self-reported health and last healthcare visit omitted from this table. For the unabbreviated results table, see Supplementary File. Statistical significance was defined as *P* < .05 in two-tailed tests.

* *P* < .05.

** *P* < .01.

*** *P* < .001.

As shown in Table 3, across all three types of data use, female respondents were less comfortable compared to male respondents. Age was inconsistently associated with comfort, whereby adults 45 and older were less comfortable than 18- to 29-year-olds with the use of health-related and sensitive data types. We also observe that non-Hispanic White respondents’ average comfort with the use of personal characteristic and health-related data is higher than that of non-Hispanic Black respondents, holding all else constant. This may indicate a racial inequality in the uses of data for prediction in healthcare.

## Discussion

This analysis captures public comfort with data use for prediction in healthcare. Analyzing a national survey of US adults, we find that while comfort with data use for prediction is generally low, the public is comparatively more comfortable with the use of more overtly clinical data (blood pressure, cancer diagnoses) than demographic or social data types. The data collected and used by health systems to make predictions about patients are not restricted to overtly clinical data types. Prediction in practice involves the use of widely varied data types like religious identity and whether patients have unpaid medical bills.^11^^,^^12^ This study finds that the system of prediction is developing in a way that could undermine trust by violating public comfort and expectations.

We observe higher comfort with the use of health-related data than personal characteristic data for prediction. Public comfort was lowest with the use of sensitive data types (eg, income, history of incarceration) for prediction in healthcare. There was little variation in comfort with the use of this type of data, with 87.6% of respondents indicating discomfort. It should be noted that some data types traditionally considered sensitive for the purposes of data privacy and protection (eg, genetic information, mental health status) do not load on factor 3 with the sensitive data types identified in this analysis. Prior work indicates differences between public comfort on the one hand and current policy for data protection on the other. For example, a national survey found no significant differences between public desire for notification related to the use of their health data compared to their biospecimens, which does not reflect current policy distinctions between the two.^33^ Similarly, our study indicates a disparity between what policy defines as sensitive as compared to what the public perceives as sensitive. If the use of these sensitive data can be avoided in predictive modeling without sacrificing model quality, that could be responsive to public concerns. However, because sensitive data types can be useful for prediction in certain cases, transparency and communication may also ameliorate some threats to patient trust when they must be used, as suggested by previous studies examining desire for notification with respect to information use. For example, conveying why income is important for the quality of a specific predictive model might ameliorate patient concerns. It may also indicate competence with data use and bolster trust.

This work reveals three multivariate factors of public comfort with use of data for prediction. These factors and their internal consistency are confirmed using multiple methods and demonstrate conceptual coherence: (1) personal characteristic data, (2) health-related data, and (3) sensitive data. Multivariable analysis identifies that respondent sex, perception of system competence in data use, and perceived system integrity in data use are consistently positively associated with comfort with all three categories of data for prediction. Experiences of discrimination in healthcare are negatively predictive of comfort with the use of personal characteristic data and sensitive data use. As health systems increasingly implement new AI-driven predictive models, additional investment in health equity by addressing discrimination in patient care and ensuring transparency about the use of data as well as the predictive models being used in clinical care may facilitate patient comfort. These results highlight the importance of relationships between patients and their health systems in public attitudes about predictive technologies and data use.

There are limitations to this study that should inform interpretation. First, sexual identity is limited to binary self-identified sex in the dataset, which is an incomplete measure. The relationships between gender and comfort with data use for prediction are thus not presented here. Second, Native American and Alaskan Native respondents are not identified in this sample. This is a limitation in the data’s representativeness and future work should ensure that Native American and Alaskan Native respondents are (1) specifically identified and (2) included in analysis of comfort with data use. It is possible that the explanatory video introduces some degree of cognitive bias, but was necessary to provide a baseline understanding of the survey topic for respondents. The survey questions analyzed here do not measure comfort with data use for specific types of predictive models, which may vary. For example, people may feel more negatively toward the use of data on mental health diagnoses for predicting bill payment than for predicting stroke. For other data types, like history of incarceration or income, there may be less variation in comfort across predictive model applications. Future work should compare reported comfort with data use in general, as we have done, and for specific predictive models to identify whether comfort differs by predictive model application. Qualitative exploration of why people feel concerned about specific data types, and under what circumstances, will further explain these findings and their implications.

## Conclusion

This analysis reveals that comfort with the use of data for prediction in healthcare is generally low among US adults. Because predictive models in healthcare draw on increasingly diverse data types, and because known models have explicitly included some of the data types identified as unacceptable to a large majority of participants in a national sample, we find misalignment between public comfort and current practice and the need to reconsider risks of data sharing beyond identifiability. Building trust and engaging the public about the use of data in predictive models is important for the quality, sustainability, and patient-centeredness of the healthcare system as predictive technologies expand.

## Supplementary Material

ocae009_Supplementary_Data

## Data Availability

Data available on request.
