# Supplementary material for: Public perspectives on the use of different data types for prediction in healthcare
Source: J Am Med Inform Assoc. 2024 Feb 1;31(4):893–900. doi: 10.1093/jamia/ocae009 (PMC10990535; doi:10.1093/jamia/ocae009)
Supplement: ocae009_Supplementary_Data [file ocae009_supplementary_data.docx]

**Supplementary File**

**Description of predictive models**

| Predictive models use information about you, and other people like you, to predict what you might want or do in the future. For example, Netflix predicts what you might watch based on the movies you have already watched. Amazon looks at what you have bought, and what other people buy, to guess what you might buy next.  Predictive models are also used in healthcare. They can help a doctor anticipate if you are at high risk of an allergic reaction. They can help identify the most effective treatments for you based on information about you and other patients like you. Predictive models can also help health systems decide how many patients to schedule based on whether people have missed their appointments in the past. |
| --- |

**Key term definitions**

| My healthcare system  "Your healthcare system" refers to the healthcare professionals and institutions that you personally interact with when getting health care.  The healthcare system  "The healthcare system" refers generally to the healthcare system in this country.  Healthcare providers  Health care providers include people such as doctors and nurses who provide medical treatment.  Electronic health record  A digital version of your paper chart or medical record. An electronic health record contains your medical and treatment history including diagnoses, medications, treatment plans, immunization dates, allergies, radiology images, and laboratory and test results.  Health information  Health information includes information about you and your medical treatment history including diagnoses, medications, treatment plans, immunization dates, allergies, radiology images, and laboratory and test results.  De-identified [health information or biospecimens]  De-identified means that "identifying information" about you is removed from your health information. Identifying information includes things like your name, address, date of birth, etc. |
| --- |

**NORC Methodology**

**Factor Analysis and Cronbach’s Alpha**

Eigen values indicate that factors 1-3 should be retained, which is confirmed by the Scree plot (Figure S1). The eigen value of Factor 1 was 8.43. Factor 2 was 1.34 and Factor 3 was 0.69. Cumulative variance explained by the three factors is approximately 100%. For additional information on the scree test, see Cattell RB. The Scree Test for the Number of Factors. Multivariate Behavioral Research. 1966 Apr;1(2):245–76. For details on factor analysis and the Kaiser-Meyer-Olkin threshold, see Shrestha N. Factor Analysis as a Tool for Survey Analysis. AJAMS. 2021 Jan 20;9(1):4–11.

Figure S1. Scree plot

Cronbach’s alpha measures for each factor, created according to factor loadings for the full sample, were high and well above the acceptability threshold of 0.65 (Table S1). This confirms that the three multivariate factors identified in the full sample are valid. With the full sample, Cronbach’s alpha for each identified factor was high (at least 0.88) and well above the acceptability threshold of 0.65. This confirmed the validity of the multivariate factors identified in the exploratory factor analysis.

Table S1. Measures of internal consistency for each multivariate factor

| Factor | Cronbach’s α for full sample | Items |
| --- | --- | --- |
| 1 | 0.93 |  |
|  |  | Age |
|  |  | Sex |
|  |  | Race |
|  |  | Weight |
| 2 | 0.92 |  |
|  |  | Cancer diagnosis |
|  |  | Blood pressure |
|  |  | Health behaviors |
|  |  | Mental health |
|  |  | Genetic information |
| 3 | 0.90 |  |
|  |  | Income |
|  |  | Marital status |
|  |  | Employment status |
|  |  | History of incarceration |
|  |  | Zip code |
|  |  | Insurer |
|  |  |  |

This analysis identifies three multivariate factors of public comfort with use of various data types for prediction in healthcare. The factors are robust and confirmed through multiple statistical tests. The multivariate factors are found to be valid across all racial and ethnic groups represented in this dataset.

**Multivariable analysis**

Table S2. Full results of multivariable OLS regression of each multivariate factor of comfort on independent variables of interest and all covariates

|  | Personal characteristic data | | Health-related data | | Sensitive data | |
| --- | --- | --- | --- | --- | --- | --- |
|  | b | p-value | b | p-value | b | p-value |
|  |  |  |  |  |  |  |
| Female (ref. male) | -0.21 | <0.001*** | -0.13 | 0.002** | -0.16 | <0.001*** |
| Age |  |  |  |  |  |  |
| 18-29 | ref |  | ref |  | ref |  |
| 30-44 | 0.003 | 0.97 | -0.16 | 0.07 | -0.13 | 0.14 |
| 45-59 | -0.13 | 0.21 | -0.27 | 0.004** | -0.29 | <0.001*** |
| 60+ | -0.14 | 0.17 | -0.22 | 0.02* | -0.32 | <0.001*** |
| Race/ethnicity |  |  |  |  |  |  |
| White | ref |  | ref |  | ref |  |
| Black | -0.19 | 0.004** | -0.16 | 0.01* | 0.002 | 0.97 |
| Hispanic | -0.02 | 0.71 | 0.003 | 0.96 | 0.06 | 0.26 |
| Asian | 0.04 | 0.8 | 0.03 | 0.83 | 0.15 | 0.2 |
| Other | -0.16 | 0.18 | -0.08 | 0.48 | 0.08 | 0.39 |
| Education |  |  |  |  |  |  |
| Less than high school | ref |  | ref |  | ref |  |
| High school | -0.24 | 0.1 | -0.13 | 0.34 | -0.03 | 0.8 |
| Some college | -0.18 | 0.2 | -0.07 | 0.6 | -0.02 | 0.84 |
| BA or more | -0.05 | 0.75 | 0.04 | 0.73 | 0.01 | 0.93 |
| Annual household income |  |  |  |  |  |  |
| <$50,000 | ref |  | ref |  | ref |  |
| At least $50,000 | 0.1 | 0.03* | 0.1 | 0.02* | -0.03 | 0.45 |
| Health insurance coverage |  |  |  |  |  |  |
| No | ref |  | ref |  | ref |  |
| Yes | 0.2 | 0.03* | 0.12 | 0.16 | 0.07 | 0.34 |
| Self-reported health |  |  |  |  |  |  |
| Poor to fair | ref |  | ref |  | ref |  |
| Good | -0.03 | 0.57 | -0.05 | 0.32 | -0.01 | 0.77 |
| Very good to excellent | -0.02 | 0.74 | -0.08 | 0.17 | 0.03 | 0.55 |
| Last healthcare visit |  |  |  |  |  |  |
| Longer than one year ago | ref |  | ref |  | ref |  |
| Within past year | -0.04 | 0.53 | -0.05 | 0.44 | 0.001 | 0.99 |
| Self-reported health |  |  |  |  |  |  |
| Poor to fair | ref |  | ref |  | ref |  |
| Good | -0.03 | 0.58 | -0.06 | 0.22 | -0.01 | 0.78 |
| Very good to excellent | -0.03 | 0.68 | -0.09 | 0.11 | 0.03 | 0.59 |
| Last healthcare visit |  |  |  |  |  |  |
| Longer than one year ago | ref |  | ref |  | ref |  |
| Within past year | -0.03 | 0.66 | -0.05 | 0.45 | 0.01 | 0.93 |
| Experienced discrimination |  |  |  |  |  |  |
| No | ref |  | ref |  | ref |  |
| Yes | -0.13 | 0.03* | -0.02 | 0.69 | -0.13 | 0.008** |
| System integrity with data | 0.08 | 0.01* | 0.09 | <0.001*** | 0.11 | <0.001*** |
| System competence with data | 0.35 | <0.001*** | 0.36 | <0.001*** | 0.27 | <0.001*** |

**Weighted multivariable analysis**

This supplementary analysis includes the same multivariable models as those presented in the body of the manuscript with the general population weights applied. Thus, the results presented in this table are national representative estimates. Because the unweighted sample included oversamples of African American respondents, Hispanic respondents, and respondents earning less than 200% of the federal poverty level, the influence of the perspectives of these groups in the table below are down-weighted.

|  | Personal characteristic data | | Health-related data | | Sensitive data | |
| --- | --- | --- | --- | --- | --- | --- |
|  | b | p-value | b | p-value | b | p-value |
|  |  |  |  |  |  |  |
| Female (ref. male) | -0.14 | 0.07 | -0.03 | 0.67 | -0.12 | 0.1 |
| Age |  |  |  |  |  |  |
| 18-29 | ref |  | ref |  | ref |  |
| 30-44 | 0.07 | 0.61 | -0.11 | 0.44 | -0.14 | 0.15 |
| 45-59 | -0.06 | 0.67 | -0.2 | 0.16 | -0.28 | 0.01* |
| 60+ | -0.05 | 0.73 | -0.13 | 0.32 | -0.31 | 0.001** |
| Race/ethnicity |  |  |  |  |  |  |
| White | ref |  | ref |  | Ref |  |
| Black | -0.17 | 0.08 | -0.15 | 0.07 | -0.01 | 0.9 |
| Hispanic | -0.08 | 0.46 | -0.03 | 0.73 | 0.02 | 0.87 |
| Asian | 0.11 | 0.46 | 0.14 | 0.34 | 0.05 | 0.68 |
| Other | -0.27 | 0.05 | -0.14 | 0.41 | 0.08 | 0.44 |
| Education |  |  |  |  |  |  |
| Less than high school | ref |  | ref |  | ref |  |
| High school | -0.22 | 0.21 | -0.09 | 0.67 | -0.05 | 0.81 |
| Some college | -0.08 | 0.68 | 0.02 | 0.94 | 0.01 | 0.94 |
| BA or more | -0.004 | 0.98 | 0.1 | 0.64 | 0.02 | 0.94 |
| Annual household income |  |  |  |  |  |  |
| <$50,000 | ref |  | ref |  | ref |  |
| At least $50,000 | 0.11 | 0.18 | 0.12 | 0.08 | -0.02 | 0.66 |
| Health insurance coverage |  |  |  |  |  |  |
| No | ref |  | ref |  | ref |  |
| Yes | 0.25 | 0.03* | 0.08 | 0.34 | 0.05 | 0.58 |
| Self-reported health |  |  |  |  |  |  |
| Poor to fair | ref |  | ref |  | ref |  |
| Good | -0.06 | 0.5 | -0.04 | 0.52 | -0.01 | 0.85 |
| Very good to excellent | -0.06 | 0.53 | -0.1 | 0.18 | 0.02 | 0.75 |
| Last healthcare visit |  |  |  |  |  |  |
| Longer than one year ago | ref |  | ref |  | ref |  |
| Within past year | 0.07 | 0.31 | 0.05 | 0.48 | -0.01 | 0.8 |
| Experienced discrimination |  |  |  |  |  |  |
| No | ref |  | ref |  | ref |  |
| Yes | -0.16 | 0.07 | -0.04 | 0.48 | -0.11 | 0.06 |
| System integrity with data | 0.08 | 0.02* | 0.07 | 0.03* | 0.1 | 0.01* |
| System competence with data | 0.31 | <0.001*** | 0.36 | <0.001*** | 0.28 | <0.001*** |
